# Small area contextual effects on self-reported health: Evidence from Riverside, Calgary

**DOI:** 10.1186/1471-2458-10-264

**Published:** 2010-05-20

**Authors:** Jenny Godley, Valerie A Haines, Penelope Hawe, Alan Shiell

**Affiliations:** 1 Dept. of Sociology, University of Calgary, Calgary, Canada; 2 Dept. of Community Health Sciences, University of Calgary, Calgary, Canada; 3 Population Health Intervention Research Centre, University of Calgary, Calgary, Canada

## Abstract

**Background:**

We study geographic variation within one community in the City of Calgary using a more fine-grained geographic unit than the Census tract, the Census Dissemination Area (DA). While most Riverside residents consider their neighbourhood to be a fairly cohesive community, we explore the effect of socio-economic variation between these small geographic areas on individuals' self-reported health, net of individual level determinants.

**Methods:**

We merge data from the 2001 Census for Riverside, Calgary with a 2004 random telephone survey of Riverside residents. Our data are unique in that we have information on individuals from every DA wholly contained in the Riverside community. These data enable us to conduct multinomial logistic regression analyses of self-reported health using both individual-level and DA-level variables as predictors.

**Results:**

We find significant variation in measures of DA socio-economic status within the Riverside community. We find that individual self-reported health is affected by variation in an index of DA-level socio-economic disadvantage, controlling for individual variation in gender, age, and socio-economic status. We investigate each aspect of the DA index of disadvantage separately, and find that average education and the percent of households that are headed by a lone parent are most important.

**Conclusions:**

These findings demonstrate that, even within a cohesive community, contextual effects on health can be located at a smaller geographic level than the Census tract. Research on the effects of local area socio-economic disadvantage on health that combines administrative and survey data enables researchers to develop more comprehensive measures of social and material deprivation. Our findings suggest that both social and material deprivation affect health at the local level.

## Background

There is evidence showing the effects of place of residence on health, over and above individual characteristics [1-3]. Empirical evidence demonstrates that local socio-economic disadvantage has a negative impact on many objective measures of health status, including mortality [4,5], cardiac disease [6], obesity [7,8], depression [9], and maternal and child health [10,11]. Additionally, much research shows the negative impact of neighbourhood disadvantage on health care utilization [12], and on subjective measures of self-reported health, controlling for individual-level variables [13,14]. This paper focuses on the effects of place of residence on self-reported health, which is generally considered to be a good overall measure of adult health and is predictive both of illness and of health care utilization [15].

Much of the Canadian and American literature on contextual effects on health uses administrative data to measure the characteristics of place of residence [16]. The geographic boundaries are often set at the level of the Census tract, since Census tract level data are most readily available to social science researchers, and most easily merged with survey data. The Census tracts, which contain between 2,500 and 8,000 people (in the 2001 Census, Census tracts in Calgary contained an average of 4,687 people), are then defined as the 'neighbourhoods' by researchers [17,18]. However, evidence suggests that setting the boundary of the neighbourhood by the Census tract may mask significant local variation that can be tapped only by using data on smaller contextual units [19,20].

Census Dissemination Areas (DAs, formerly called Enumeration Areas in Canada) contain approximately 125-440 dwellings, or 400-700 people, and are the smallest unit for which Canadian Census data are publicly available. Recognizing the limitations of defining neighbourhoods using Census tract boundaries, some Canadian health researchers use DA-level data to model neighbourhood effects on health [6-8,11,12]. However, many of these authors still aggregate the DA-level data to create comparison groups of 'low-income neighbourhoods' and 'affluent neighbourhoods.'

We address the effects of place of residence on health in one unique community in Calgary, Riverside (a pseudonym). Residents of Riverside (which is defined by the City of Calgary as a 'neighbourhood,') have a long history of community pride. Riverside is a community of Calgary with low socio-economic status. It occupies a picturesque part of the city, by the river, and was for a long time a separate village with its own identity and governance [21]. While the homes in one or two of the streets nearest the water and away from the main roads are identifiably middle class, most people live in small houses or condominiums, and there is high transient population (especially in the areas concentrated in and around a trailer park) [22]. The research partnership with the University, within which this study is embedded, began with a request from the United Way to investigate any possible foundation for Riverside's strong reputation for community strength and pride in Calgary.

While outsiders may see Riverside as a fairly disadvantaged community, insiders feel a strong sense of community pride. In our 2004 survey of Riverside residents (described below), when asked, "Do you think of Riverside as your community?" 81% of survey respondents said yes. And when asked, "Would you say that you feel 'at home' in Riverside?" 97.5% said yes. Given that Riverside appears to be such a socially meaningful and cohesive community, we are interested in examining how much socio-economic variation there is within Riverside. Additionally, we examine whether such variation has an impact on self-reported health for Riverside residents.

Utilizing a unique dataset which contains data on individuals within every DA that is wholly contained within Riverside, this paper examines the following questions to contribute to the literature on contextual effects on health: (1) Do the DAs that make up the community of Riverside differ in their socio-economic characteristics? (2) Is DA socio-economic disadvantage associated with self-rated health for residents of Riverside, net of attributes of residents? (3) Which aspects of DA disadvantage are most important in predicting self-reported health, net of individual level characteristics?

## Methods

This paper combines data from the 2001 Census of Canada with data from a 2004 survey of Riverside residents. A list of random phone numbers was generated, and households were contacted first by letter and then by telephone. The interviewers asked to speak with the member of the household who was 18 or over and had the most recent birthday. Of the 762 households contacted by telephone, 441 completed the survey, generating a response rate of 58%.

Census data were examined first at the city and community level, and then at the Census Dissemination Area level [23]. Community district boundaries are determined by the City of Calgary; Statistics Canada provides Census data at this community level for the City. There are seven Census Tracts which are at least partially contained within Riverside's boundaries (as defined by the City of Calgary). There are 26 DAs which lie completely within the boundaries of Riverside, and 6 DAs which lie partially within Riverside's boundaries. For the purposes of this paper, we examine only the DAs that lie completely within Riverside's boundaries.

Data from the Census were merged into the Riverside survey data using the 2005 Postal Code Conversion File (PCCF) provided by Statistics Canada. Each respondent to the survey supplied his or her household address and postal code. The PCCF links the six-character postal code with the standard 2001 Census geographic areas - dissemination areas, census tracts, and census subdivisions. Using the postal codes provided by the respondents, we merged data from the Census at the Dissemination Area (DA) into the survey data.

### Individual-level Measures

Our dependent variable is self-reported health. Self-reported health was measured using the following question "In general, would you say that your health is excellent, very good, good, fair or poor?"

Independent variables measured at the individual level include self-reported gender, employment status, age, and education. Educational attainment was coded into four categories (less than high school, high school certificate, post-secondary trade or diploma, and bachelor's degree or higher), and we use this measure as a proxy for social class. Respondents were asked to place themselves in an age group (18-24; 25-34; 35-44; 45-54; 55-64; 65+). Employment status was coded 1 if employed and 0 if unemployed or retired.

### DA-level Measures

For the DA-level measures, we examined data from the 2001 Census of Canada. We used the following census variables: median family income; the incidence of low income (the percent of economic families or unattached individuals within the area who are below the low income cut-offs, as defined by Statistics Canada) [24]; the percent lone parent households; and average education. For the aggregate education measure, we created a summary measure of educational attainment by multiplying the percent less than high school, high school graduate, post-secondary diploma, attended university and BA or higher by 1, 2, 3, 4, and 5 respectively and summing the results.

## Results

Table 1 presents descriptive data on the characteristics of the 2004 Riverside survey respondents, compared to the same characteristics measured in the 2001 Census for Riverside [23], (boundaries defined by the City of Calgary), and for Calgary as a whole.

**Table 1:** Sample characteristics.

Characteristics	Sample^a^	Riverside^b^	Calgary^c^
N	441	11480	871140
Gender			
Percent Male	40	50	50
Age group (%) (only 18+ included)			
18-24	6	9	11
25-34	16	22	23
35-44	19	25	24
45-54	26	20	19
55-64	15	11	11
65+	18	13	12
Employment			
Percent Employed	68	68	72
Education (%)			
Less than High School	15	28	21
High School	26	23	19
Technical/Diploma	37	39	37
University +	21	10	23
Missing	1		
Self-Reported Health (%)			
Excellent	23.0	N/A	N/A
Very good	36.0		
Good	26.0		
Poor/Fair	14.5		
Missing	.5		

^a ^Data from the Riverside survey, 2004

^b ^Data from the City of Calgary, compiled from the Census of Canada 2001

^c ^Data from the Census of Canada 2001

In terms of educational attainment, Riverside residents are less likely to have university degrees than residents of Calgary as a whole. However, sample respondents are more likely to have university degrees than residents of Riverside as a whole. Overall, survey respondents are older than average Riverside and Calgary residents (since the survey was administered to those 18 and over, we only include data for age groups 18 and over in Table 1). The sample is 40% male and 68% of respondents are employed. The sample distribution for the dependent variable, self-reported health, is also shown in Table 1.

Table 2 presents data from the 2001 Census of Canada at different levels of aggregation - for Calgary as a whole, for Riverside (as bounded by the City of Calgary), and for the 26 Census Dissemination Areas in Riverside [23]. The education value for Calgary, 4.01, indicates that on average Calgarians have some university education. The average values for the DAs in Riverside range from 3.01, slightly over post-secondary diploma, to 4.40, in between some university education and a BA or higher.

**Table 2:** Socio-economic variation within Riverside DAs (N = 26), compared to Calgary.

Geographic Unit	Average Self-Reported Health^a^	Median Family Income^b^	Percent Low Income^b^	Average Education^b^	Percent Lone Parent Households^b^	Index of Disadvantage
**Calgary**	NA	57,879	11	4.01	15	NA
						
**Riverside**	NA	40,468	18	3.49	24	NA
						
**Census DA**						
106	2.92	37,416	24.40	3.31	40.00	0.98
112	3.05	30,703	39.40	3.34	33.33	1.38
126	3.14	39,880	31.40	3.01	28.57	1.12
117	3.29	44,690	15.00	3.25	37.14	0.52
101	3.30	43,665	11.80	3.57	39.47	0.20
116	3.50	59,393	24.10	3.52	10.34	-0.55
109	3.50	54,315	11.00	3.54	30.00	-0.32
103	3.56	62,723	14.70	3.59	33.33	-0.42
110	3.57	44,438	32.70	3.27	19.05	0.56
107	3.59	56,722	17.40	3.41	10.34	-0.55
120	3.60	54,697	22.70	3.48	11.11	-0.39
125	3.64	50,723	7.80	3.98	26.09	-0.82
105	3.67	56,539	24.40	3.53	40.00	0.23
113	3.67	29,385	59.20	3.38	27.59	1.78
108	3.70	57,937	21.40	3.19	20.00	-0.03
121	3.75	NA	NA	3.90	23.08	NA
115	3.79	65,781	17.10	3.96	16.67	-1.19
104	3.85	30,573	35.20	3.80	33.33	0.83
102	3.89	48,540	13.70	3.41	20.00	-0.19
119	3.89	38,706	24.00	3.33	29.03	0.66
114	3.90	29,776	32.60	3.35	18.92	0.88
118	3.93	44,009	24.50	3.26	10.64	0.16
111	3.94	44,853	36.40	3.53	25.93	0.55
122	3.98	70,041	7.00	4.40	5.41	-2.27
124	4.00	82,008	14.00	4.25	12.66	-2.11
123	4.09	59,055	17.10	3.95	16.67	-1.00
						
Mean	3.64	49,463	23.16	3.56	24.00	0.00
						
**Coefficient of Variation**	**8.33%**	**27.15%**	**50.88%**	**9.48%**	**43.42%**	**NA**

^a ^Data from 2004 Riverside Survey

^b ^Data from 2001 Census

Relative to the rest of Calgary, Riverside is a disadvantaged community. Yet there appears to be variation in disadvantage across the DAs within Riverside. To capture the variation in DA-level disadvantage within Riverside in our models of self-reported health, we first create a summary measure of disadvantage for each DA [25]. While there is no standard way to calculate a place-based index of deprivation, most authors agree that such an index should include measures of both material and social deprivation [26-28]. Our index of socio-economic disadvantage is the result of a factor analysis that combines the four indicators of median income, incidence of low income, percent lone-parent households, and average education for each DA. Later, we disaggregate this index to examine each of the indicators separately.

Table 2 provides evidence that the answer to our first research question is yes. There is considerable variation across the 26 DAs on all of the socio-economic characteristics. The coefficients of variation are all above 1%, rising to 50.88% for percent low income and 43.42% for percent lone parent households. For each of the indicators, there are some DAs that score well above the Calgary average and there are some that score well below the Calgary average. We note that the mean values for the 26 DAs of median family income, incidence of low income, and average education do not match the values for Riverside as a whole because the unit of analysis in this table is the DA. These means do not control for differences in population between DAs.

The index of disadvantage ranges from -2.27 to 1.78 (mean and median = 0, standard deviation = 1, inter-quartile range = 1.2). The factor loadings are as follows: median income .921; incidence of low income .766; percent lone parent households .601; and average education .793. Cronbach's alpha is .72. A high value on this index indicates high disadvantage, while a low value indicates low disadvantage.

Additionally, there is variation amongst respondents to our survey across the 26 DAs in Riverside on our dependent variable, self-reported health. The DAs are listed from low to high average self-reported health, which ranges from 2.92 (between 'fair' and 'good') to 4.09 (between 'very good' and 'excellent'). The coefficient of variation for this variable is 8.33%.

Having established variation within the Riverside community on both our dependent and our DA-level independent variable, we ran two multinomial logistic regression models to predict self-reported health. First, we regressed self-reported health on four individual-level predictor variables: age, gender, employment status and education. Next, we re-ran the regression model including the DA index of disadvantage. Results are shown in Tables 3 and 4, below. In both models, poor/fair health is used as the reference category for the dependent variable.

**Table 3:** Multinomial logistic regression results for self-reported health: Individual-level variables.

	Good vs. Poor/Fair		Very Good vs. Poor/Fair		Excellent vs. Poor/Fair	
	*B*	O.R. (95% CI)	*B*	O.R. (95% CI)	*B*	O.R. (95% CI)
Gender	-.636*	0.529 (0.271-1.035)	-.559	.572 (0.297-1.101)	-.470	.625 (0.310-1.259)
Age 18-24	-.085	0.918 (0.171-4.924)	.567	1.763 (0.382-8.145)	.789	2.202 (0.471-10.283)
Age 25-34	.345	1.412 (0.458-4.355)	.038	1.038 (0.355-3.036)	-.043	.958 (0.313-2.931)
Age 35-44	.382	1.466 (0.498-4.318)	.137	1.147 (0.409-3.214)	.108	1.114 (0.383-3.239)
Age 45-54 (reference category)	---		---		---	
Age 55-64	.469	1.599 (0.572-4.471)	-.409	.664 (0.232-1.907)	-.387	.679 (0.226-2.039)
Age 65+	.520	1.681 (0.580-4.871)	.814	2.256 (0.758-6.486)	-.319	.727 (0.215-2.455)
Less than High School (reference category)	--		--		--	
High School	.725	2.064 (0.844-5.046)	1.757**	5.793 (2.152-15.596)	1.444**	4.236 (1.443-12.434)
Technical	.220	1.246 (0.547-2.838)	1.71***	5.546 (2.239-13.739)	1.239*	3.451 (1.266-9.407)
University Degree	.342	1.408 (0.453-4.371)	2.22***	9.209 (2.930-28.947)	2.141**	8.507 (2.540-28.487)
Employed	1.152**	3.164 (1.408-7.109)	1.80***	6.028 (2.645-13.736)	1.479**	4.390 (1.870-10.307)
						
Intercept	-.362		-1.55**		-1.359*	

Model Fit:

Chi-square (DF = 30) 89.237***

Pseudo R-square^a ^0.184

*Notes: *N = 439; Beta coefficients (B) and *Odds Ratios (O.R.) *shown.

*p < .05, ** *p *< .01 ***p < .001 (2-tailed tests). ^a ^Cox and Snell Pseudo R-squared reported.

**Table 4:** Multinomial logistic regression results for self-reported health: Including DA-level disadvantage.

	Good vs. Poor/Fair		Very Good vs. Poor/Fair		Excellent vs. Poor/Fair	
	*B*	O.R. (95% CI)	*B*	O.R. (95% CI)	*B*	O.R. (95% CI)
DA disadvantage	-.283	0.754 (0.528-1.076)	-.211	.809 (0.571-1.147)	-.503**	.605 (0.418-0.874)
Gender	-.723*	0.485 (0.246-0.959)	-.634	.531 (0.274-1.029)	-.521	.594 (0.291-1.213)
Age 18-24	-.103	0.902 (0.169-4.809)	.530	1.698 (0.373-7.740)	.956	2.601 (0.558-12.131)
Age 25-34	.447	1.563 (0.501-4.876)	.020	1.020 (0.343-3.031)	.232	1.261 (0.403-3.950)
Age 35-44	.474	1.606 (0.542-4.760)	.206	1.229 (0.436-3.464)	.330	1.390 (0.470-4.115)
Age 45-54 (reference category)	---		---		---	
Age 55-64	.545	1.724 (0.600-4.953)	-.384	.681 (0.229-2.023)	-.295	.744 (0.236-2.349)
Age 65+	.451	1.569 (0.528-4.663)	.893	2.443 (0.826-7.225)	-.293	.746 (0.205-2.711)
Less than High School (reference category)	--		--		--	
High School	.622	1.863 (0.751-4.626)	1.696**	5.454 (2.000-14.869)	1.374*	3.949 (1.311-11.901)
Technical	.089	1.093 (0.473-2.526)	1.572**	4.817 (1.922-12.069)	1.033*	2.809 (0.998-7.903)
University Degree	-.036	0.964 (0.297-3.136)	2.004**	7.416 (2.296-23.954)	1.705*	5.502 (1.574-19.236)
Employed (reference category)	1.085*	2.959 (1.296-6.756)	1.80***	6.053 (2.593-14.132)	1.474**	4.366 (1.793-10.630)
Intercept	-.186		-1.46*		-1.39*	
						
*Model Fit:*						
Chi-square (DF = 33)	96.889***					
Pseudo R-square^a^	0.202					

*Notes: *N = 439; Beta coefficients (B) and *Odds Ratios (O.R.) *shown.

*p < .05, ** *p *< .01 ***p <. 001 (2-tailed tests).

^a ^Cox and Snell Pseudo R-squared reported.

Examining the results in Table 3, we find that in this sample, self-reported health is significantly affected by respondents' education and employment status. Controlling for gender, age and education, respondents who are employed are more likely to report good, very good or excellent health rather than poor/fair health. And controlling for gender, age and employment, those with a high school education or above are more likely to report very good or excellent health rather than poor/fair health compared to those with less than a high school education. Overall, we explain about 18% of the variance in self-reported health using the variables age, gender, employment status, and education level.

The results in Table 4 demonstrate that including the DA index of disadvantage improves our explanation of self-reported health, as we increase our explanatory power to 20% of the variance. The index of disadvantage is also significantly related to individuals' self-reported health, controlling for individual-level variables. An individual who lives in a dissemination area with higher socio-economic disadvantage is less likely to report excellent health rather than poor/fair health, controlling for age, gender, employment status and education.

Next, we disaggregated the index of disadvantage and examined the effects of each of the individual components of the index (median income, percent low income, percent lone parent households, and average education) on self-rated health, net of the individual level variables. Results from these analyses are not included in the paper, but are available from the authors upon request. We found that as independent variables, neither median income nor percent low income had an effect on self-reported health, net of individual level variables. Percent lone parent households and average education did have an effect, though.

Individuals who lived in DAs with higher average levels of education were more likely to report excellent, rather than poor/fair health, controlling for age, gender, individual level education and employment. Individuals who lived in DAs with a higher percentage of lone parent families were more likely to report poor/fair health rather than very good or excellent health, net of the individual level variables.

## Discussion

Using Census data, we find substantial variation within a socially cohesive community on indicators of socio-economic disadvantage. We find that the index of disadvantage at the Census DA level predicts self-reported health, controlling for individual-level variables. Our findings suggest that local area does have an impact on health, net of individual-level variables.

Once we disaggregate the index of disadvantage, our findings further emphasize the importance of understanding the effects of both material and social deprivation at the local level. In particular, we find that at the DA level, average education and percent lone parent families are both predictors of individual-level health. In their work developing a deprivation index using Census data, Pampalon et al use six indicators from which they derive two dimensions of deprivation - material deprivation (education, employment, and income) and social deprivation (marital status, living along, and single parent families) [29]. Following this typology, we interpret our findings to suggest that at the DA level, both material and social deprivation (as indicated by average education and percent lone parent families) are associated with individual-level health.

Survey data enables researchers to develop measures of material and social disadvantage that complement what is available in the Census [30]. In our survey, we attempted to capture social deprivation through questions on community cohesion. We found that these measures did not vary across DA, with respondents consistently reporting high levels of community attachment (low levels of social deprivation). The Census measures of disadvantage did vary across DA, though, and did predict self-rated health. This study demonstrates the usefulness of combining survey and Census data to measure local area disadvantage, especially within cohesive communities.

We acknowledge that our results are limited by our sample. The response rate for the survey was low (58%), and we know that the respondents differ from average Riverside residents both in terms of age and education. Somewhat surprisingly, gender and age do not affect self-rated health in our individual level models (with the exception of gender in the good versus poor/fair comparison). This unusual finding is probably explained by the non-representative gender and age composition of our sample, and by the fact that age was measured as a categorical, rather than a continuous, variable.

We do not have enough cases within each DA to use hierarchical linear modeling techniques, thus we have not accounted for possible heterogeneity across DAs in our regression models. We acknowledge that since our data are clustered, standard errors for the regression coefficients may be underestimated. Thus our findings should be interpreted cautiously. Furthermore, we are limited by the fact that we are merging Census data collected in 2001 with survey data collected in 2004. We do not know if the Riverside residents in our sample resided in the same DA in 2001 and 2004.

We emphasize the unique nature of Riverside as a community, and argue that this study provides a first step by demonstrating that even within a cohesive neighborhood, small area geographic effects on self-reported health, although modest, do exist. However, we also acknowledge that we do not know that the variation within Riverside is typical of other communities within Calgary or indeed communities in other cities.

## Conclusions

Future research should examine multiple communities within a city, and eventually across cities in Canada. Ideally, future research would have a larger sample size within the DAs within each community, to enable multi-level analysis. This would enable researchers to address all three levels of geographic effects on health evidenced in the Census data: household, DA, and Census tract, as suggested by Tranmer and Steel (2001) [31]. Additionally, as Kwan (2009) suggests, future research on neighborhood effects should examine how much time people actually spend in their neighborhood of residence [32].

Recently, researchers have moved from demonstrating contextual effects on health to speculating about the pathways and mechanisms through which these contextual effects operate [19,33,34]. Our findings of DA-level effects of disadvantage on health within a single cohesive community, net of individual-level predictors, invite consideration of which area-level mechanisms might be more potent at small units of analysis. Bernard et al (2007) suggest that social resources (informal networks, local sociability, and community organizations) may be more important than material resources in terms of access to health related resources at the local level [35].

We find that levels of material and social deprivation both vary across DAs, and both affect self-reported health, net of individual characteristics. Future studies of these small area contextual effects on health should continue to combine survey data with Census data. Using these complementary data sources, researchers can develop robust measures of disadvantage to examine the separate effects of material and social deprivation on health.

## Competing interests

The authors declare that they have no competing interests.

## Authors' contributions

JG conducted the data management and analysis, interpreted the results, and drafted the manuscript. VAH assisted with research questions, interpretation of results, and manuscript preparation and revision. PH conceived of the study, designed and supervised data collection procedures and helped to revise the manuscript. AS assisted with statistical analysis, interpretation of results, and manuscript revision. All authors read and approved the final manuscript.

## Pre-publication history

The pre-publication history for this paper can be accessed here:

http://www.biomedcentral.com/1471-2458/10/264/prepub
